# MANAGEMENT OF ERYTHEMA NODOSUM LEPROSUM BY MYCOPHENOLATE MOFETIL

**DOI:** 10.4103/0019-5154.43205

**Published:** 2008

**Authors:** Kalyan Banerjee, Raghubir Banerjee

**Affiliations:** *From the University College of Medicine, Goenka Hospital, Calcutta, India*

**Keywords:** *Erythema nodosum leprosum*, *mycophenolate mofetil*

## Abstract

Mycophenolate mofetil has been tried in 20 cases of chronic relapsing erythema nodosum leprosum reaction where long use of systemic steroid produce complications or are contraindicated. Excellent results have been observed in all the cases to arrest the reaction followed for a period of six to eight months duration.

## Introduction

Progressive knowledge of leprosy over the last few decades sometimes fall short in the understanding of many a subsequent complication and aftermath of this disease even after a full multidrug therapy (MDT) schedule. Treatment of reactional and post reactional episodes often pose a therapeutic challenge to leprologists of our country and some times drug therapy with routine anti-inflammatory and immunomodulator agents fail to produce desired response.^1^

The present study attempts to evaluate the role of a yet not so routinely used agent Mycophenolate Mofetil in reactions in leprosy where systemic corticosteroid therapy is not viable.

## Materials and Methods

In this study profile 20 patients of erythema nodosum leprosum (ENL) between the age group of 30 to 50 years were selected out which 16 patients were of lepromatous leprosy (LL) type and 4 patients of borderline lepromatous leprosy (BL) type. These patients had intractable ENL reactions of severe grade refractory to routine conservative treatment and having contraindications for systemic corticosteroid therapy like hypertension, diabetes mellitus and peptic ulcer and history of melena.

All the patients had erythematous evanescent, tender skin nodules with pyrexia and joint pains as the commonest clinical findings.^2^ Routine laboratory tests showed leucocytosis and raised erythrocyte sedimentation rate (ESR). In four patients skin biopsy was done for confirmation of the diagnosis. Fever and nodules and erythema subsided within 48 to 72 h of commencement of therapy.

These patients were started with Mycophenolate Mofetil at dose of 1 gram per day and in a few cases dosage increment to 2 grams per day were necessary for control and subsequent maintenance. The liver function, kidney function and routine blood counts were done during the course of therapy. These patients were monitored for a period of six to eight months. In two patients treatment was discontinued within a month due to the systemic side-effects of the drug like nausea and vomiting and other gastro intestinal symptoms and drug did not produce desired results. Maintenance dosages of 500 mg per day was effective to control the ENL in most cases.

## Results

Out of the 20 cases under going treatment 14 cases were males and 6 females mostly of the middle age group. Most of the cases (18) showed significant subsidence of lesions within a month although low dose maintenance with 0.5 gram per day was necessary for 4 to 6 months for complete remission Table 1.

**Table 1:** Showing number of patients with different contra indication to systemic steroid therapy

Contraindications	No of patients
Hypertension	7
Diabetes mellitus	5
Peptic ulcer	6
Melaena	2

## Discussion

The name ENL given by Japanese Dr. Murata 1912 is used in most parts of Asia alternatively lepromatous lepra reaction is also used as it commonly occurs at the bacilliferous end of the leprosy spectrum. It is common in treated lepromatous patients 50% developing by the end of the first year of treatment.^3^

Mycophenolate Mofetil is used. In dermatology and documented in the treatment of SLE, pemphigus, steroid resistant idiopathic thrombocytopenic purpura and other autoimmune diseases. Its use in ENL as a steroid sparing agent has not so commonly being put to test. Efficacy of Mycophenolate Mofetil in diffuse proliferative lupus nephritis and in stable renal transplant recipients has been commonly put to practice by the medical fraternity.^4^,^5^

Immunopharmacology of Mycophenolate Mofetil is vital in understanding its clinical usage. Prodrug Mycophenolic acid acts as a inhibitor of inosine mono phosphate dehydrogenase (IMPDH) which is the rate limiting enzyme in denovo synthesis of guanosine nucleotide. T and B lymphocytes are more dependent on this path way that alters cell types. Mycophenolic acid (MPA) is five times more potent inhibitor of type (ii) isoform of IMPDH which is expressed in activated lymphocytes than type (i) isoform. MPA has cytostatic effect on lymphocytes than on other cell types (mechanism by which MPA exerts immunosuppressive effects).

Efficacy of MPA occurs mainly due to induction of apoptosis of activated T cells, eliminating clones of cells responding to antigenic stimulation. Depleting guanosine nucleotides, MPA suppresses glycosylation and expression of some adhesion molecules. By depleting guanosine nucleotides, MPA also depletes tetrahydrobiopterin (cofactor for inducible form of nitric oxide synthetase - INOS).

The common dosage schedule followed was the routine 0.5 gram twice in a day with dosage reduction over period of 4 to 6 months and further follow-up for another two months.

## Conclusion

Mycophenolate Mofetil may be an important steroid sparing agent for ENL reactions where systemic cortisone is contraindicated for producing quick control of symptoms and remission lasting over 6 to 8 months.

## References

[1] Karat AB, Chatterjee BR (1978). Complications of leprosy, a window on leprosy.

[2] Thangaraj RH (1983). Reactions in leprosy, A manual of leprosy.

[3] Waters MF, Chatterjee BR A window on leprosy.

[4] Bakr MA, Gheith OA, Ismael AM (2008). Rescue immunosuppressive therapies in living-related renal allotransplant: a long-term prospective randomized evaluation. Exp Clin Transplant.

[5] Sampaio EL, Pinheiro-Machado PG, Garcia R, Felipe CR, Park SI, Casarini DE, Moreira S, Franco MF, Tedesco-Silva H, Medina-Pestana JO (2008). Mycophenolate mofetil vs sirolimus in kidney transplant recipients receiving tacrolimus-based immunosuppressive regimen. Clin Transplant.

